# Pediatric invasive fungal rhinosinusitis

**DOI:** 10.3389/fped.2023.1090713

**Published:** 2023-04-25

**Authors:** Perla Villamor, Valeria Arango, Cristina Cortes, Carlos de la Torre

**Affiliations:** ^1^ Pediatric Otolaryngologist, Hospital Serena del Mar, Cartagena, Colombia; ^2^ Hospital Infantil Napoleón Franco Pareja, Cartagena, Colombia; ^3^ Otolaryngology Resident, Faculty of Medicine, Universidad de Cartagena, Cartagena, Colombia; ^4^ Pediatric Otolaryngologist, Hospital Infantil de México Federico Gómez, México City, México

**Keywords:** pediatric invasive fungal rhinosinusitis, diagnosis, prognosis, clinical algorithm, invasive fungal disease

## Abstract

Pediatric invasive fungal rhinosinusitis (PIFR) is a rapidly progressive, potentially fatal disease. Previous medical literature demonstrates that its early diagnosis significantly reduces the risk of mortality in these patients. This study aims to present an updated clinical algorithm for optimized diagnosis and management of PIFR. A comprehensive review was conducted with only original, full-text articles published in English and Spanish from Cochrane Library, Pub-Med/MEDLINE, Embase, Scopus, and Google Scholar between January 2010 and June 2022. Relevant information was extracted and then integrated to develop a clinical algorithm for a proper diagnosis and management of PIFR.

## 1. Introduction

Pediatric invasive fungal rhinosinusitis (PIFR) is an infection affecting immunocompromised patients with high morbidity and mortality rates. PIFR is a devastating submucosal infiltration of fungal pathogens in the nasal cavity and paranasal sinuses. In this pathology, saprophytic fungi become pathological under immunosuppressive circumstances. These organisms lead to rapidly progressive angioinvasion and necrosis, spreading to adjacent structures like the orbit and the brain (1–3).

A delay in surgical management is associated with high mortality, especially in children, due to its rapid orbital and cerebral extension. A timely diagnosis with a opportune surgical debridement followed by systemic antifungal therapy is crucial to increase survival (4). Therefore, the main objective of this study was to present an updated review and clinical algorithm for early diagnosis of PIFR.

Epidemiological data in the pediatric population is limited. Based on the available literature, the estimated incidence is 1.5–2 per 1,000 pediatric patients with an underlying hematological disease (1). Advancement and standardization for PIFR treatment protocols are limited due to the rarity of the disease, resulting in a lack of published randomized studies. The present study reviewed the most recent available literature to define a protocol for early diagnosis and management in children. The suggested algorithm begins with the early identification of high-risk patients.

The algorithm will allow clinicians and surgeons to approach pediatric patients at high risk of invasive fungal sinusitis in a fast and structured way improving opportune diagnosis and treatment to decrease the risk of sequelae, morbidity, and mortality.

**Keywords:** Rhinosinusitis, Fungal Infection, Pediatrics, Immunosuppression.

## 2. Materials and methods

A comprehensive literature review was performed using the Cochrane Library, PubMed/MEDLINE, Embase, Scopus, and Google Scholar databases. The search was limited to articles published in English and Spanish. Literature from January 2010 to June 2022 was reviewed using a dual-prong search to capitalize on MeSH terms, subheadings, and free text/keywords. The titles and abstracts of retrieved articles were reviewed and selected by two authors (V.P and A.V). Single case reports, or material focused on non-invasive forms of fungal sinusitis were excluded. Full-text review was performed by both authors to assure the studies included documented evidence of pediatric invasive fungal sinusitis, information on the diagnostic methods used, details of medical and surgical treatments received, and survival outcomes.

## 3. Results

This review produced 262 articles in the identification phase. An initial screening of abstracts excluded 204 studies. Discarded studies were mostly single case reports, or material focused on non-invasive forms of fungal sinusitis. The remaining 58 articles underwent full-text review, 43 of which were subsequently excluded for studying other forms of fungal disease (non-invasive, pulmonary, systemic, etc.).

Collected results are summarized in a clinical algorithm, with its pillars consisting mainly in identifying high-risk patients, screening this population, performing of complementary studies, and starting treatment in the shortest possible time, improving the prognosis of patients.

### 3.1. High-risk patients

Over the past decade, the incidence of invasive fungal infections in children has increased because of more aggressive oncologic treatments implemented to increase patient survival. The main scenario for high-risk patients with PIFR is a pediatric oncology unit. 90% of patients with PIFR have a diagnosis of hematologic malignancy, primarily acute myeloid leukemia (5). Approximately 1%–2% of hospitalized patients with hematologic disorders will present a PIFR (6). Patients at highest risk are those with prolonged severe neutropenia, defined as a count less than 500 mm^3^ for more than 10 days, and/or prolonged corticosteroid therapy at a dose of 0.5 mg/kg per day for more than 30 days (6). However, this information comes from studies in adults, so its generalization to children should be done caution. Recently, Muayad Alali et al. (7) developed a risk score for pediatric invasive fungal disease (IFD) based on odds ratios for the following dichotomous risk factors: duration of fever (greater than 4.5 days: +5), duration of neutropenia (more than 9.5 days: +2), hypotension (5th percentile blood pressure adjusted for age or delay in capillary refill >3 s: +1), age group, (greater than 8.5 years: +1), absolute lymphocyte count (less than 250: +2) and absolute monocyte count (greater than 100: −1) resulting in a risk score of 1–11 for each patient (Table 1). Setting a score ≤5 as the optimal cut-off point to discriminate low-risk patients with a specificity of 0.73 and a cut-off point to discriminate high-risk patients with a score of 8, with a sensitivity of 0.93 (Table 2) (7).

**Table 1:** Risk score for pediatric invasive fungal disease.

Risk Factor	Score
Presence of Febrile Neutropenia	1
Fever duration ≥4.5 days	5
Neutropenia duration ≥9.5 days	2
Hypotension	1
Age ≥8.5 years	1
Absolute lymphocyte count <250	2
Absolute monocyte count >100	−1
**Overall Score**	**11**

**Table 2:** Patient risk score interpretation.

Score	Risk % of Invasive Fungal Disease (IFD)	Interpretation	
1	0.01%	LOW RISK	**Low Risk** Pediatric oncologic patients with this score have a risk of 1 in 1,000 (score of 1) to 1 in 140 (score of 5) of developing IFD.
2	0.03%		
3	0.1%		
4	0.2%		
5	0.7%		
6	1.8%	MODERATE RISK	**Moderate Risk** Pediatric oncologic patients with this score have a 1 in 55 (score of 6) to 1 in 21 (score of 7) risk of developing IFD.
7	4.7%		
8	12%	HIGH RISK	**High Risk** Pediatric oncologic patients with this score have a 1 in 8 (score of 8) to 3 in 4 (score of 11) risk of developing IFD
9	27%		
10	50%		
11	73%		

Other risk factors reported are the presence of septal deviations, which represent 18 times greater risk of PIFR, as well as summer months with 65 times greater risk with statistically significant differences that seem to be related to higher average and maximum temperatures compared to other seasons of the year (8).

### 3.2. Lethality and survival

PIFR is a highly lethal disease, with a mortality rate of approximately 40%–50%. The invasive nature of the illness is limitless, and structures around the nasal cavities such as the nervous system and the orbit are no exception (6). Patients who suffer from PIFR usually have systemic comorbidities and are in poor or general conditions. A study of pediatric and adult patients showed that fever occurs in 93% of patients but it is a nonspecific systemic symptom, while localized symptoms are generally not evident until the disease is advanced (9). Survival depends on an early diagnosis for opportune antifungal therapy and debridement (4).

Other factors that appear to impact survival include improving neutropenia in high-risk patients by granulocyte transfusion of colony-stimulating factors. Green et al. found there was no significant difference in absolute neutrophil count (ANC) at the time of diagnosis between the survival and the mortality groups. However, there was a significant difference between the survival and the mortality subgroups in the recovery of ANC at a mid-treatment interval (midpoint through antifungal therapy) with mean ANC being 4,290.5/mm^2^ and 169/mm^2^, respectively (*P* < 0.001). Survival was associated with an improvement of ANC to within normal limits, while patients within the mortality group had no recovery of their ANC at the time of decease (10).

### 3.3. Screening

The clinical diagnosis of PIFR is defiant due to both its unpredictable symptomatology and nonspecific imaging findings. Often, patients present with fever and sinonasal symptoms as nasal obstruction, nasal crusts, rhinorrhea, facial edema, and/or palatal ulceration (4). Hospitals with pediatric hematology units should include accurate screening protocols to evaluate for PIFR among at-risk patients. Patients with moderate and high-risk scores for invasive fungal risk (Tables 1, 2) will benefit from screening for the timely detection of PIFR.

**Figure 1:**
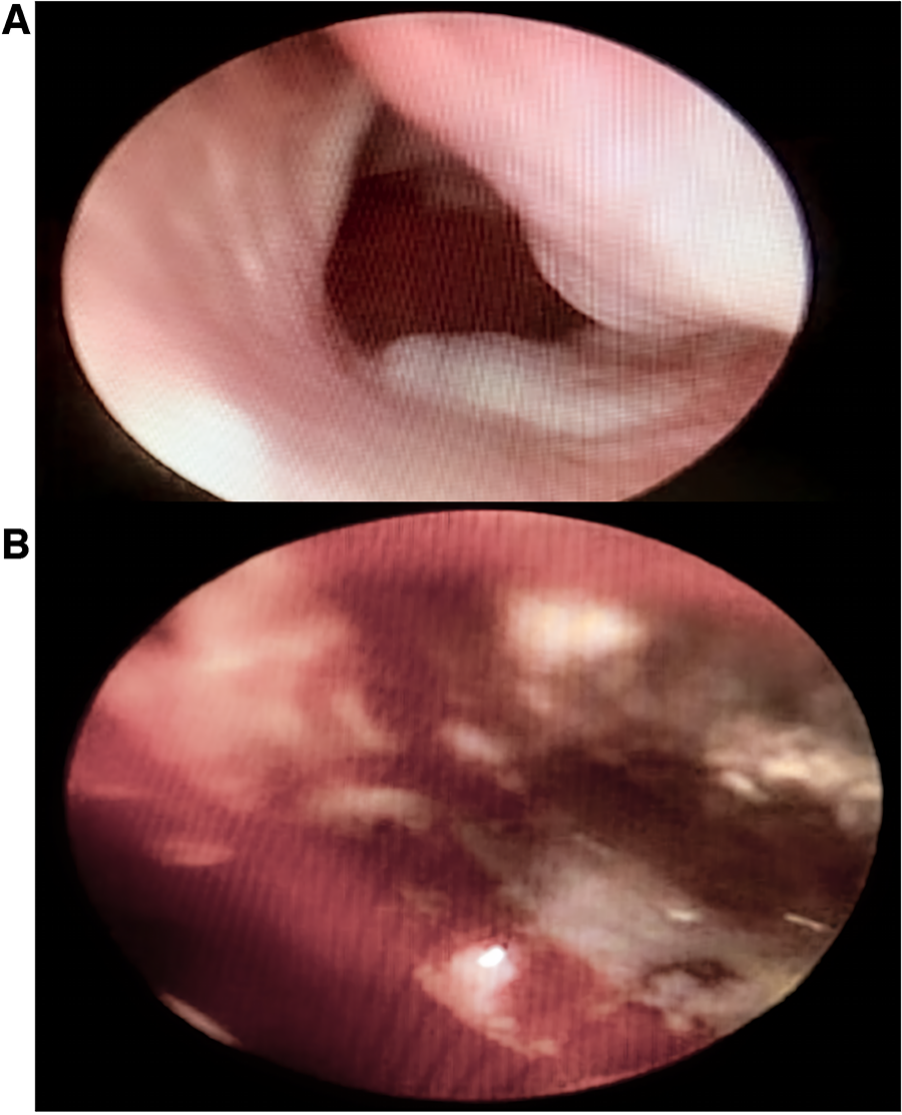
Clinical images of endoscopic appearances of pediatric invasive fungal rhinosinusitis. Typical findings on endoscopy are pale/greyish nasal mucosa (**A**) and bloody crusts (**B**).

Bedside nasal endoscopy is possible among pediatric patients and may be a sensitive tool in the diagnosis (12). Nasal endoscopy is an essential diagnostic tool as it allows the identification of mucosal changes, which are the most consistent and specific clinical findings in PIFR (12). The site of nasal compromise is variable, and thus identification of mucosal changes on endoscopy is necessary to guide biopsies (13). Mucosal discoloration, ulceration, necrosis, granulation, and reduced sensation are present in up to 75% of adults with invasive fungal sinusitis. Typical findings on rhinoscopy and endoscopy are pale/greyish nasal mucosa and bloody crusts (Figure 1).

Mulvey et al. found no patient with normal or non-concerning bedside endoscopic exam in patients with PIFR (11). Additionally, the site of nasal involvement is variable, and therefore, identification of mucosal changes at endoscopy is necessary to guide any biopsy (12).

### 3.4. Diagnostic tests

#### 3.4.1. Radiologic images

Radiological findings of invasive fungal sinusitis concerning on bone destruction are best evaluated with computed tomography (CT). However, bone destruction appears comparatively late in the disease process. Accordingly, sinus CT findings may be nonspecific and underestimate the extent of the disease, especially in the pediatric population (14). More recent studies suggest using early magnetic resonance imaging (MRI) scanning in patients with suspected invasive fungal sinusitis based on the possibility of underestimation of disease with CT. One of the fundamental advantages of MRI over CT is that it has better soft-tissue contrast resolution allowing it to detect subtler initial manifestations of the infiltrative nature of this aggressive infection, particularly the development of soft-tissue abnormalities outside the limits of the paranasal sinuses. These findings are remarkably found anterior or posterior to the maxillary sinus walls. Groppo et al. found that MRI was more sensitive than CT for the diagnosis of acute invasive fungal sinusitis (sensitivity 85%–86% compared with 57%–69%), extra sinus invasion with MRI was the most sensitive individual factor (87% and 100%), and loss of contrast enhancement agreed with endoscopic mucosal findings 76.5% of the time (15).

#### 3.4.2. Microscopy

Documentation of fungal elements in fresh clinical specimens and histological sections demonstrates infection. Micromorphology may provide information on the fungal class, however, microscopy does not definitively distinguish *Aspergillus* from other filamentous fungi (10). *Aspergillus* shows dichotomous and septate hyphae, whereas Mucorales present with septate and 90° angle branching hyphae. The distinction between septate (e.g., *Aspergillus*) and non-septate hyphae (e.g., Mucorales) is relevant as it may affect the selection of antifungal management (16).

During a bedside nasal endoscopy, it is possible to take samples of nasal secretions or a mucous smear for a microscopic examination with 10% KOH, which may reveal the presence of hyphae. Demonstrating invasion of the tissue by hyphae through microscopic examination is a proven diagnosis of invasive fungal infection (16).

#### 3.4.3. Culture

Histopathologic examination of biopsy specimens demonstrating mucosal invasion by fungal organisms and cultures are the gold standard for diagnosis, showing the specific etiologic agent and allowing antifungal susceptibility testing. However, it is not sensitive enough and can take several days to achieve a positive result. Samples obtained by brushing or deep-site tissue biopsies should be grown on Sabouraud-dextrose agar (SDA), brain-heart-infusion agar (BHI), or potato-dextrose agar (PDA) at 30°C and 37°C for 72 h. Blood cultures are usually negative, while cultures of respiratory tract secretions lack high sensitivity since *Aspergillus* is cultured from sputum in only 35% of patients with an active infection. Isolation of *Aspergillus* species from respiratory samples may indicate infection, allergy, colonization, or contamination (17).

Culture is crucial in mucormycotic diagnosis, but will be positive in only 50% of cases, even if hyphae are evident in microscopy (18). Retrospective autopsy studies have shown that only 50% of patients who have had disseminated invasive candidiasis had positive blood culture results (10). In children, blood cultures also present a low sensitivity (<50% for *Candida* spp., <5% for *Aspergillus* spp, and between 10%–40% for *Fusarium* spp) (19).

#### 3.4.4. Polymerase chain reaction (PCR)

In adults, *Aspergillus* PCR has a combined sensitivity of 84% and specificity of 76%, but when true-positive cases required two previous positive PCR results, the specificity increased to 94%. In children, *Aspergillus* PCR has sensitivities that range from 63% to 100% depending on the study, specific patient population, and PCR assay selection (20). For non-Aspergillus invasive fungal disease, microarray analyses have a sensitivity of 64% and a specificity of 80%, detecting 15 different types of fungi (*Aspergillus*, *Candida*, *Fusarium*, Mucor, Rhizopus, *Scedosporium*, and *Trichosporon* species). The best results reported are a combination of DNA-microarray with specific PCR for *Aspergillus* in biopsy samples, obtaining a sensitivity of 79% and specificity of 71% (20).

### 3.5. Management

**Figure 2:**
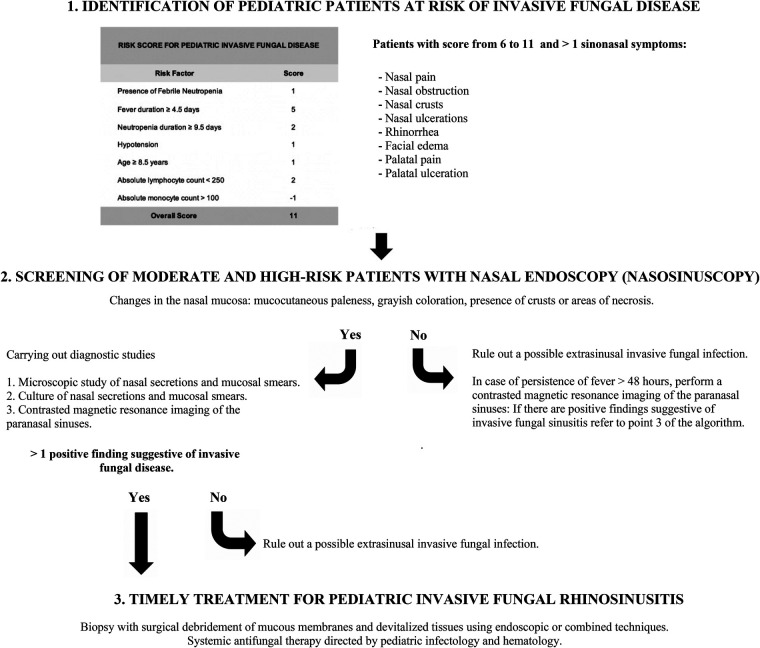
Clinical algorithm for early diagnosis and management of pediatric invasive fungal rhinosinusitis.

Surgical debridement with adjunct systemic antifungal therapy produces results of the highest quality (4, 5). Antifungal treatment has progressed in recent years with the inclusion of new agents. Amphotericin B (AmB) is now approved for patients with invasive fungal diseases and as an empirical treatment for patients with persistent neutropenia. The indications for AmB as the first line of therapy are invasive aspergillosis, mucormycosis, and invasive candidiasis (4). However, debridement is crucial when treating PIFR as necrotic tissue from angioinvasion must be removed to improve the administration of systemic antifungal therapy (6) (Figure 2).

## 4. Discussion

Epidemiological data in the pediatric population is limited. Based on the available literature, the estimated incidence is 1.5–2 per 1,000 pediatric patients with an underlying hematological disease (1). Mortality is also variable but presumed higher than in invasive mycosis in adults within the first six weeks after diagnosis (4). Advancement and standardization for PIFR treatment protocols are limited due to the rarity of the disease, resulting in a lack of published randomized studies. The present study reviewed the most recent available literature to define a protocol for early diagnosis and management in children.

The suggested algorithm begins with the early identification of high-risk patients. We found that these patients usually come from pediatric oncology units, particularly those with severe and prolonged neutropenia (5, 6). In these patients, fever and sinus symptoms, as nasal obstruction, nasal crusts, rhinorrhea, facial edema, and/or palatal ulceration (4), dictate screening with a bedside nasal endoscopy to identify any altered mucosal features. As angioinvasion of the fungus generates thrombosis, infarction, and tissue necrosis, if pale/greyish areas or bloody crusts are observed, complementary studies should be considered (11). In case of persistent fever after 48 hours, a contrasted magnetic resonance imaging of the paranasal sinuses should be performed.

Regarding diagnostic images, MRI is more sensitive than CT in diagnosing acute invasive fungal sinusitis. The most relevant findings are extra sinus invasion, especially anterior or posterior to the maxillary sinus walls, with loss of contrast enhancement (13–15). Diagnostic images provide an additional benefit in opportune surgical planning, evaluating orbital and central nervous system extension. Ruling out intracranial extension is critical as it is associated with a high mortality rate of 75%–100% despite radical surgery and antifungal therapy (21).

Adequate samples for KOH mounts, fungal culture, biopsy, and frozen section of the suspicious tissue should guide treatment (22). KOH mounts act as a screening test alone, and fungal culture, although time-consuming, provides a definitive diagnosis (23). However, fungal culture results are conclusive only in certain patients due to the reduced viability of zygomycetes hyphae (24). Therefore, empirical fungal therapy should be considered in high-risk cases with endoscopic or radiological findings suggesting PIFR.

The recovery of the absolute neutrophil count to normal levels in parallel with systemic antifungal therapy and surgical debridement of tissues is essential to improve survival. There is no specified treatment time frame reported in the literature, but rather the emphasis of initiating treatment until other factors contributing to the infection are adequately controlled, as well as clinical and microbiological improvement (25, 26). Various approaches have been described for surgical debridement, from the more traditional lateral rhinotomy including exenteration when indicated, to fully endoscopic techniques (14). However, timely and opportune debridement has proven to be more effective in improving survival rather than the type of surgical approach (4).

Although this comprehensive literature review had a small number of studies due to a lack of literature on this rare entity, to our knowledge this is the first clinical practical and optimized algorithm for diagnosis and management of PIFR. Considering the mortality rate remains high, based on the best literature available, we believe this algorithm will be useful for clinicians to ensure timely and opportune diagnosis and treatment.

## 5. Conclusions

Progress and standardization of PIFR treatment protocols are limited due to the rarity of the disease, resulting in a lack of published randomized studies. The present study reviewed the most recent available literature to define a protocol for its early diagnosis and management.

In patients with PIFR, proper diagnosis with early surgical debridement and systemic antifungal therapy will dramatically improve survival. This review offers a practical and optimized algorithm starting by identifying patients with moderate to high risk of invasive fungal disease, followed by screening with nasosinuscopy, complementary diagnostic studies, and early medical and surgical treatment to improve patient survival.
