# Supplementary material for: Pediatric invasive fungal rhinosinusitis
Source: Front Pediatr. 2023 Apr 25;11:1090713. doi: 10.3389/fped.2023.1090713 (PMC10167005; doi:10.3389/fped.2023.1090713)
Supplement: Supplementary file 3 [file Datasheet1.pdf]

1. IDENTIFICATION OF PEDIATRIC PATIENTS AT RISK OF INVASIVE FUNGAL DISEASE

RISK SCORE FOR PEDIATRIC INVASIVE FUNGAL DISEASE	
**Risk Factor**	**Score**
Presence of Febrile Neutropenia	1
Fever duration ≥ 4.5 days	5
Neutropenia duration ≥ 9.5 days	2
Hypotension	1
Age ≥ 8.5 years	1
Absolute lymphocyte count < 250	2
Absolute monocyte count > 100	-1
**Overall Score**	**11**

Patients with score from 6 to 11 and > 1 sinonasal symptoms:

- Nasal pain
- Nasal obstruction
- Nasal crusts
- Nasal ulcerations
- Rhinorrhea
- Facial edema
- Palatal pain
- Palatal ulceration

2. SCREENING OF MODERATE AND HIGH-RISK PATIENTS WITH NASAL ENDOSCOPY (NASOSINUSCOPY)

Changes in the nasal mucosa: mucocutaneous paleness, grayish coloration, presence of crusts or areas of necrosis.

Yes

Carrying out diagnostic studies

1. Microscopic study of nasal secretions and mucosal smears.
2. Culture of nasal secretions and mucosal smears.
3. Contrasted magnetic resonance imaging of the paranasal sinuses.

> 1 positive finding suggestive of invasive fungal disease.

Yes → 3.

No → Rule out a possible extrasinusal invasive fungal infection.

No

Rule out a possible extrasinusal invasive fungal infection.

In case of persistence of fever > 48 hours, perform a contrasted magnetic resonance imaging of the paranasal sinuses: If there are positive findings suggestive of invasive fungal sinusitis refer to point 3 of the algorithm.

3. TIMELY TREATMENT FOR PEDIATRIC INVASIVE FUNGAL RHINOSINUSITIS

Biopsy with surgical debridement of mucous membranes and devitalized tissues using endoscopic or combined techniques.
Systemic antifungal therapy directed by pediatric infectology and hematology.
